# Inverse association between serum lipid profiles and hepatocellular carcinoma risk: a meta-analysis of epidemiological studies

**DOI:** 10.3389/fonc.2025.1644677

**Published:** 2025-10-22

**Authors:** Chongshi Zeng, Shuran Liu, Haining Li, Xiao Han

**Affiliations:** ^1^ China Three Gorges University School of Science and Technology, Yichang, China; ^2^ College of Biological Science and Engineering, Fuzhou University, Fuzhou, China

**Keywords:** meta-analysis, liver cancer, risk, serum lipid, cholesterol, HDL, LDL, triglycerides

## Abstract

**​Background:**

The relationship between serum lipid profiles and hepatocellular carcinoma (HCC) risk remains controversial. We aimed to clarify this association through a systematic meta-analysis of epidemiological studies.

**​Methods:**

A systematic literature search was conducted in PubMed, Embase, and Web of Science (2000–May 2023) for prospective, retrospective, and cross-sectional studies reporting adjusted risk estimates (HR/OR) of HCC associated with serum lipids. Pooled effect sizes were calculated using random-effects models, with heterogeneity assessed via Cochran’s Q and I² statistics.

**​Results:**

Twenty-three studies (16 cohorts, 7 case-control) involving 1.2 million participants ((including both healthy individuals and patients with chronic liver diseases)​​) were included. Elevated serum total cholesterol (TC) was inversely associated with HCC risk (HR = 0.71, 95% CI: 0.64–0.78; I²=0%). Similar protective effects were observed for high LDL (HR = 0.46, 95% CI: 0.36–0.59; I²=97%), triglycerides (HR = 0.79, 95% CI: 0.62–0.99; I²=94%), and dyslipidemia (HR = 0.64, 95% CI: 0.50–0.83; I²=81%). No significant association was found for high-density lipoprotein (HDL). Sensitivity analyses confirmed robustness for TC and LDL, while TG results were influenced by a single study.

**​Conclusion:**

This meta-analysis provides robust evidence that elevated serum cholesterol and specific lipid subfractions are associated with reduced HCC risk. Further mechanistic studies are warranted to elucidate the role of lipid metabolism in hepatocarcinogenesis.

## 1 Introduction

Hepatocellular carcinoma (HCC) represents a significant global health burden, with epidemiological patterns demonstrating a shift from low to high sociodemographic index (SDI) regions, reflecting an etiological transition from viral to non-viral etiologies (1). Geographically heterogeneous risk profiles reveal hepatitis virus-driven carcinogenesis predominance in Asia and Africa (HBV/HCV: 75-80% of cases), contrasting with Western populations where metabolic dysfunction and genetic predisposition account for 40-50% of HCC incidence. In China, primary liver cancer ranked as the fifth most diagnosed malignancy and second leading cause of cancer mortality in 2020 (2), with established risk stratification encompassing chronic viral hepatitis (B/C), metabolic comorbidities (obesity, type 2 diabetes mellitus), behavioral factors (alcohol abuse, smoking), and aflatoxin B1 exposure (3).

The pathophysiological continuum from metabolic dysfunction-associated fatty liver disease (MAFLD) to HCC has gained prominence in industrialized nations, with MAFLD-associated cirrhosis constituting 20-30% of HCC cases in Western cohorts (4). This disease progression is mediated through insulin resistance, lipotoxicity, and chronic inflammation mechanisms, creating a pro-carcinogenic microenvironment characterized by oxidative stress and cytokine dysregulation (5). Notably, 65-80% of MAFLD-HCC cases develop in non-cirrhotic livers, underscoring the need for improved biomarkers linking metabolic dysfunction (elevated HbA1c, hypertriglyceridemia) to early hepatocarcinogenesis (6).

Experimental models demonstrate high-fat diet-induced steatohepatitis and HCC in mice through sterol regulatory element-binding protein 1 (SREBP1)-mediated lipogenesis and cholesterol crystallization pathways (7). However, clinical epidemiological data remain controversial, with pooled analyses showing paradoxical inverse correlations between serum total cholesterol (TC) and HCC risk (RR = 0.82, 95%CI:0.75-0.90) (8). Mechanistic studies suggest lipoprotein subclass-specific effects: pro-carcinogenic LDL oxidation products (oxLDL) versus anti-inflammatory HDL-associated paraoxonase 1 (PON1) activity (9). Current meta-analyses exhibit methodological limitations including heterogeneity in lipid measurement protocols (fasting vs. non-fasting) and insufficient adjustment for statin use (10).

## 2 Materials and methods

### 2.1 Search strategy

This systematic review and meta-analysis was conducted ​in accordance with the Preferred Reporting Items for Systematic Reviews and Meta-Analyses (PRISMA) guidelines and the Meta-analysis of Observational Studies in Epidemiology (MOOSE) guidelines.

Two investigators independently performed a systematic literature search across three electronic databases (Embase, PubMed, and Web of Science) for articles published between January 2000 and May 2023. The search strategy combined Medical Subject Headings (MeSH) terms and free-text keywords, including:

- ​Disease terms: “hepatocellular carcinoma,” “liver cancer,” “HCC,” “hepatic neoplasm,” “primary liver malignancy”;
- ​Exposure terms: “serum lipid,” “plasma lipid,” “blood lipid,” “total cholesterol (TC),” “HDL-C,” “LDL-C,” “triglyceride (TG),” “dyslipidemia,” “hypercholesterolemia”;
- ​Study design terms: “epidemiological study,” “cohort,” “prospective,” “retrospective,” “case-control,” “cross-sectional.”

Boolean operators (AND/OR) were utilized to link conceptual groups. The reference lists of eligible articles were manually screened to identify additional relevant studies.

### 2.2 Inclusion and exclusion criteria

Studies were selected based on the following criteria:

Inclusion Criteria:

- ​Population: Adults (≥18 years) with or without pre-existing liver disease;
- ​Exposure: Quantified serum lipid levels (TC, LDL-C, HDL-C, TG, or dyslipidemia);
- ​Outcome: Incident primary hepatocellular carcinoma (HCC) or intrahepatic cholangiocarcinoma (ICC);
- ​Study Design: Observational studies (prospective/retrospective cohort, case-control, or cross-sectional);
- ​Statistical Reporting: Adjusted hazard ratios (HR), odds ratios (OR), or relative risks (RR) with 95% confidence intervals (CI).
- Etiology/Comorbidity Reporting:​ Studies reporting baseline prevalence of established HCC risk factors where available, including:
- Chronic viral hepatitis (HBV/HCV).
- Metabolic dysfunction-associated fatty liver disease (MAFLD)/NAFLD.
- Cirrhosis.
- Type 2 diabetes mellitus (T2DM).
- Alcohol-related liver disease (ALD).
- Aflatoxin exposure (geographically relevant populations).

Exclusion Criteria:

- Duplicate publications or overlapping datasets;
- Non-original research (reviews, commentaries, conference abstracts);
- Studies lacking full-text access or insufficient data for meta-analysis;
- Animal or *in vitro* studies;
- Participants with any cancer diagnosis prior to baseline;
- Incident cancer cases identified within 1 year of baseline (applied where data permitted).

Discrepancies between reviewers were resolved through consensus discussions.

### 2.3 Data extraction

Two researchers independently extracted the following data using a standardized form:

- Study Characteristics: First author, publication year, country, study design, follow-up duration;
- ​Participant Demographics: Sample size, age, sex, baseline comorbidities (e.g., cirrhosis, viral hepatitis); Note:​​ While some primary studies excluded participants with specific infections (e.g., HCV, HBV, HIV) or alcohol abuse (**Table 1**), extracted comorbidity data reflects the reported characteristics of included cohorts across studies.
- ​Exposure Metrics: Serum lipid thresholds, measurement methods, adjustment variables;
- Outcome Data: Adjusted risk estimates (HR/OR/RR) with 95% CIs for highest vs. lowest lipid categories.

**Table 1:** Characteristics of prospective cohort studies included in meta-analysis on associations between serum fats and liver cancer.

Author, year (Reference)	Country, study design	Baseline years	Median follow-up (Years)	Age (Mean/Median, Years)	Sex	Population exclusion	Cohort size	Cases	Exposure	Exposure assessment	Outcome	Outcome assessment	Adjustment for confounding variables
Allison, 2017	U.S., MarketScan	2008	4	57.7	Both	Participants with concomitant diagnoses of hepatitis B, alcoholic liver damage, hereditary hemochromatosis, nonalcoholic fatty liver disease, nonalcoholic steatohepatitis, cirrhosis, alpha-1 antitrypsin deficiency, autoimmune hepatitis, and Wilson disease were excluded.	29583	2931	Hyperlipidemia	Serum level	Liver cancer	Medical insurance	NR
Dong, 2020	Korea, NHIS-NSC	2002	8	>20	Both	Participants with HBV, HCV, liver cirrhosis, any cancer, heavy drinking which was defined as alcohol intake ≥30 g per day in men and ≥20 g per day in women and who had missing data for BMI , smoking status, alcohol status and ALT were excluded.	467206	236	Hyperlipidemia, TC	Validated FFQ	Liver cancer	Questionnaires	Age, sex, BMI, smoking status, alcohol intake, exercise, hypertension, diabetes, total cholesterol, dyslipidaemia and ALT level.
Fasiha, 2020	U.S., VHA	2003	9	55.5	Both	Participants with any alcohol-related International Classification of Diseases (ICD)-9 codes or positive AUDIT-C scores (≥4 in men and ≥3 in women) any time before or during study follow-up. evidence of rare chronic liver disorders (hereditary hemochromatosis, primary biliary cirrhosis, primary sclerosing cholangitis, alpha-1 antitrypsin disease, or autoimmune hepatitis) were excluded.	217817	253	Dyslipidemia	Serum level	Liver cancer	Medical records	Age, sex, race/ethnicity (non-Hispanic white, non-Hispanic black, Hispanic, and other) and health care utilization measured as the number of clinic visits in the first 2 years of NAFLD index.
Hiroyasu, 2009	Japan, JPHC	1990	12.4	40–69	Both	Participants with history of cardiovascular disease were excluded.	33,368	125	Cholesterol	Serum level	Liver cancer	Medical records and/or cancer registries	Age, BMI, pack years of smoking, ethanol intake, hypertension, diabetes, hyperlipidemia medication use, total vegetable intake, coffee intake and public health center.
Jinyan, 2011	China, EHBH	2008	NR	52.45±9.85	Both	Participants with hepatitis A virus, HCV, hepatitis D virus, hepatitis E virus, human immunodeficiency virus, Epstein-Barr virus, and cytomegalovirus infection, alcohol consumption > 30 g/day, metastatic liver cancer, autoimmune liver disease, drug-related liver disease, alcoholic hepatitis, obstructive jaundice, other causes of chronic liver disease, renal inadequacy or blood diseases were excluded.	429	179	TG, LDL-C, HDL-C	Serum level	Liver cancer	Medical records	Weight, height, blood pressures, tumor size and whether there were any violations of metastasis, medical history, life style characteristics and other related information
Jiyoung, 2009	Finland, ATBC	1985	14.9	60	men	Participants with history of cancer other than nonmelanoma skin cancer or carcinoma in situ, severe angina pectoris, chronic renal insufficiency, liver cirrhosis, chronic alcoholism, anticoagulant therapy, other medical problems that might have limited long-term participation, or current use of vitamin E (>20 mg/d), vitamin A (>20,000 IU/d), or β-carotene (>6 mg/d) supplements were excluded.	29093	68	Cholesterol, HDL	Serum level	Liver cancer	Medical records and questionnaires	Age, intervention, level of education, systolic blood pressure, BMI, physical activity, duration of smoking, number of cigarettes smoked per day, saturated fat intake, polyunsaturated fat intake, alcohol consumption.
Joseph, 2022	UK, Biobank cohort	2006	7.1	NR	Both	Participants with made for prevalent cancer at recruitment, missing MetS component data, and voluntary withdrawal from the study were excluded.	366,016	112	TG, HDL-C	Serum level	Liver cancer	National cancer and death registries	Total physical activity, height, alcohol consumption frequency, smoking intensity, frequency of red and processed meat consumption, highest educational level, regular aspirin or ibuprofen use , ever use of hormone replacement therapy and, where necessary, fasting time.
Ju, 2020	U.S., Mayo Clinic	2006	3.8	61.5	Both	Participants with HCC at initial evaluation or within the first 6 months were excluded.	354	30	Hyperlipidemia	Serum level	Liver cancer	Medical records	NR
Myung, 2020	Korea,SMC	2008	7.2	47.2	Both	Participants with younger than 18 years, co-infection with hepatitis C virus or human immunodeficiency virus, evidence of other previous or concurrent malignancy, history of HCC before the index date, development of HCC within 6 months from the index date, follow- up duration of less than 6 months, case with missing value on baseline serum cholesterol level were excluded.	7713	702	Cholesterol	Serum level	Liver cancer	Medical records	Age, sex, liver cirrhosis, comorbidities (DM and hypertension), serum HBV DNA levels, AL T level, total cholesterol, and medications (antiviral drugs and antiplatelets)
Paul, 2017	Sweden, Swedish AMORIS	1985	20.03	44	Both	Participants with benign liver tumors, primary liver cancer or cirrhosis at baseline were excluded.	509,436	766	Cholesterol, TG, HDL cholesterol, LDL cholesterol	Serum level	Liver cancer	Linkage with Swedish national registries	Age, sex, SES, triglycerides, cholesterol, raised glucose, diabetic status and history of liver disease (Cholesterol not adjusted for total cholesterol; HDL-C, LDL-C not adjusted for triglycerides).
Te-Sheng, 2022	China, Taiwan	2003	6	56.9	Both	Participants with aged <40 years, data lacking ≥70% of laboratory variables, data missing both HBV and HCV viral markers, data lacking written informed consent were excluded.	43,545	35	TG	Serum level	Liver cancer	Linkage with the mortality and cancer registration data	Age, sex, BMI, AST, TG, AFP, FIB4, GLU
Tetsuya, 2011	Japan, NHO	2002	1.3	63.2	Both	NR	337	32	Cholesterol	Serum level	Liver cancer	Medical records	Age, sex, total bilirubin, ALT, AFP ,albumin, total cholesterol
Wegene, 2012	Europe, Me-Can cohort	2006	12	44	Both	Participants with malignant cancer before the health examination were excluded.	578,700	266	Cholesterol	Serum level	Liver cancer	National cancer registries	Age, smoking status, cohort, birth year and sex, BMI.
Xiangming, 2021	China, Kailuan Cohort Study	2006	11.47±1.87	51.81±12.66	Both	Participants with a lack of TC or FBG data, a history of cancer,the administration of statins were excluded.	98,936	388	Cholesterol	Serum level	Liver cancer	Medical records and questionnaires	Age, sex, BMI, serum TC, FBG, ALT, Hs-CRP, triglycerides (TG), serum total bilirubin (Tbil), hepatitis B surface antigen positive (HBSAg(+)), cirrhosis, smoking, drinking, exercise, fatty liver, and education degree.
Yuri, 2021	Korea, NHIS	2009	7.3	57.8±11.2	Both	Participants with missing data, pre-existing cancer, taking statin medication, subjects taking statin at baseline were excluded.	8,528,790	26,891	Cholesterol, TG, LDL-C	Serum level	Liver cancer	Questionnaires and health examination data, clinical diagnosis and pharmacy	Age, sex, alcohol consumption, smoking history, physical activity, income, BMI, hypertension, DM, and fenofibrate medication.
Yuri, 2022	Korea, NHIS	2009	7.3	55.11	Both	Participants with missing data and those with preexisting viral hepatitis, liver cirrhosis, or cancer diagnoses were excluded.	1,564,597	9,372	TG, HDL-C	Serum level	Liver cancer	Questionnaires, anthropometric measurements, laboratory tests	Age, sex, smoking history, alcohol consumption, physical activity, and BMI.

Missing statistical parameters were calculated using RevMan 5.3 (Cochrane Collaboration). Corresponding authors were contacted via email to request unreported data; unresponsive inquiries were documented.

### 2.4 Risk of bias assessment

Study quality was evaluated using the Newcastle-Ottawa Scale (NOS) for observational studies, assessing three domains:

- Selection Bias: Representativeness of cohorts, exposure ascertainment;
- Comparability: Adjustment for confounders (e.g., age, sex, viral hepatitis);
- Outcome Assessment: Follow-up duration, outcome verification.

Each domain was scored as “low,” “moderate,” or “high” risk of bias. Randomized controlled trials (RCTs), if present, would have been assessed via Cochrane Risk of Bias Tool 2.0 (RoB 2).

### 2.5 Quality assessment

The methodological quality of the included cohort and case-control studies was assessed using the Newcastle-Ottawa Scale (NOS). ​As recommended by the MOOSE guidelines,​​ studies with a NOS score ≥ 7 were considered high quality.

### 2.6 Data dissection

Conducting the meta-analysis involved utilizing the RevMan 5.3 software, with all study flexibles represented as binary variables indicated by relative risk and corresponding 95% confidence intervals (11). To address potential heterogeneity among studies, the calculation of the comprehensive impact magnitude was executed using the random-effects framework. Cochran’s Q and I^2^ statistics were employed to evaluate heterogeneity. In instances where the P>0.1, signifying homogeneity across multiple studies, the fixed-effects model was implemented (12). Conversely, if P ≤ 0.1, suggesting statistically significant heterogeneity, the random-effects model was utilized. Moreover, I^2^ > 50% indicated substantial heterogeneity, prompting further evaluation of result stability through sensitivity analysis (13).

### 2.7 Sensitivity analysis

Result stability was assessed by removing the study with the maximum weight and observing the resulting change in the effect quantity.

### 2.8 Evaluation report deviation

a funnel diagram will be employed when there are at least 6 articles included, in order to examine the presence of publication bias.

### 2.9 Ethical review and informed consent from patients

The substance of this article does not necessitate moral sanction or ethical examination, and its dissemination will occur through printed materials or relevant meetings (14).

## 3 Results

### 3.1 Document characteristics

**Figure 1:**
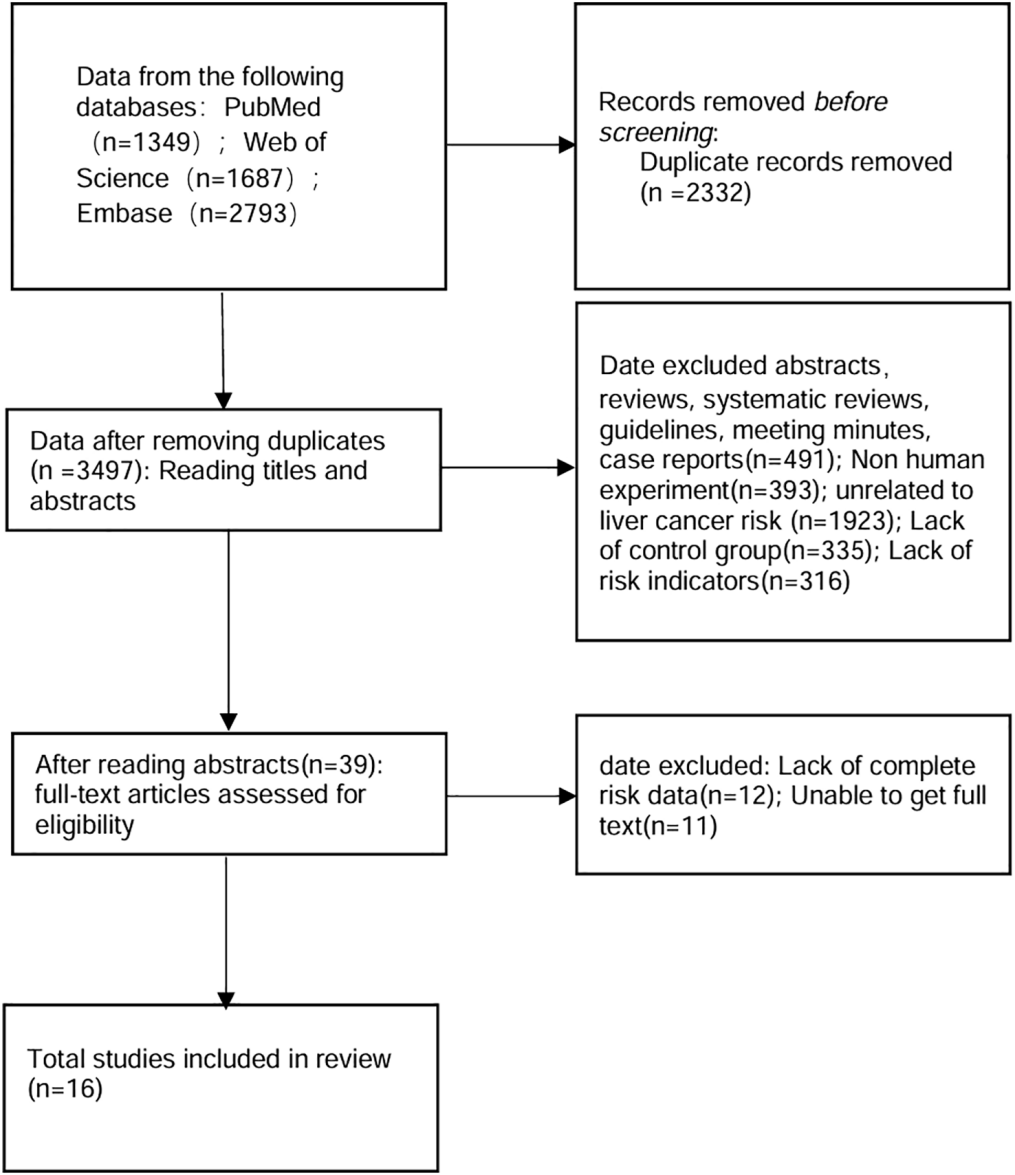
Follow chart of study selection.

After eliminating duplicates, a search across Web of Science, PubMed and Embase yielded 3497 records. Further full-text review was required for 39 articles initially identified, resulting in the exclusion of 23 articles. Ultimately, 16 prospective, retrospective, and cross-sectional studies—encompassing both healthy individuals and patients with liver-related diseases—were encompassed in the present meta-analysis (**Figure 1**, **Table 1**). Among the 16 included studies, 13 (81.3%) excluded participants with pre-existing cancer diagnoses, while 7 (43.8%) further excluded incident HCC cases occurring within 1 year of lipid measurement to address reverse causality concerns (**Table 1**) (15–30).

### 3.2 Thorough exploration into the correlation between blood lipids and the occurrence of hepatic carcinoma

**Figure 2:**
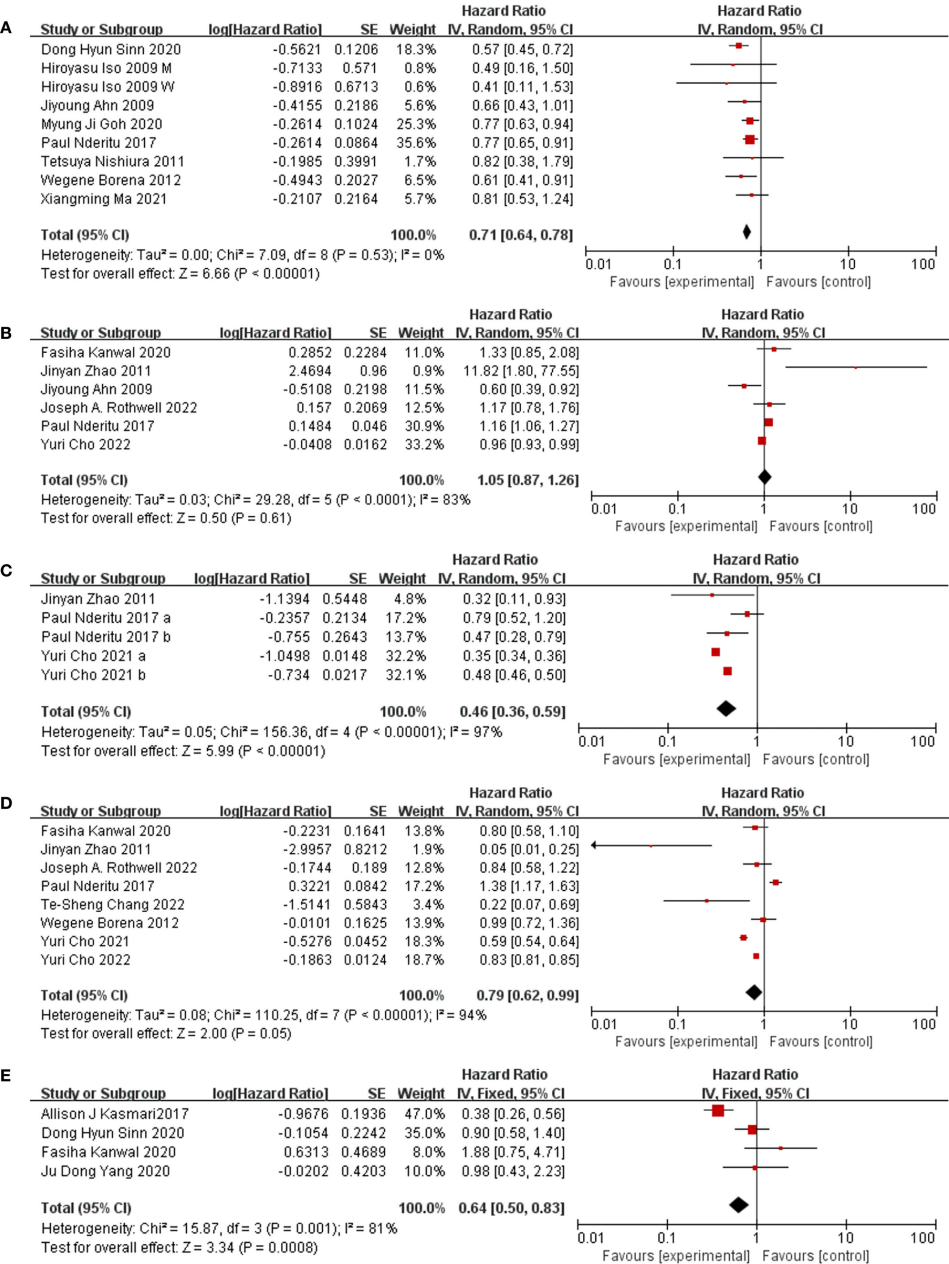
Forest plot of association. **(A)** Forest plot of association between TC and liver cancer. study people of different genders which showed as “M” (male) and “W” (female) in figure. **(B)** Forest plot of association between HDL and liver cancer. **(C)** Forest plot of association between LDL and liver cancer. study LDL of different levels which showed as “a” (high levels) and “b” (higher levels) in figure. **(D)** Forest plot of association between TG and liver cancer. **(E)** Forest plot of association between dyslipidemia and liver cancer.

Through our examination, a statistically significant connection emerged, demonstrating the association between the risk of liver cancer and the concentrations of total cholesterol in the bloodstream (HR = 0.71, 95% CI: 0.64, 0.78; I^2^ = 0%) (**Figure 2A**). Delving into cholesterol subtypes, serum HDL did not exhibit a significant effect on liver cancer occurrence. Meanwhile, serum LDL appeared to be more intricately linked to liver cancer risk than serum HDL (HR HDL = 1.05, 95% CI: 0.87, 1.26; I^2^ = 83%; HR LDL = 0.46, 95%CI: 0.36, 0.59; I2 = 97%) (**Figures 2B** and 2C). Additionally, we observed that serum triglycerides (HR = 0.79, 95%CI: 0.62, 0.99; I^2^ = 94%) (**Figure 2D**) and dyslipidemia (HR = 0.64, 95%CI: 0.50, 0.83; I^2^ = 81%) (**Figure 2E**) also showcased a declining correlation pattern concerning the risk of liver cancer.

### 3.3 Quality evaluation

**Figure 3:**
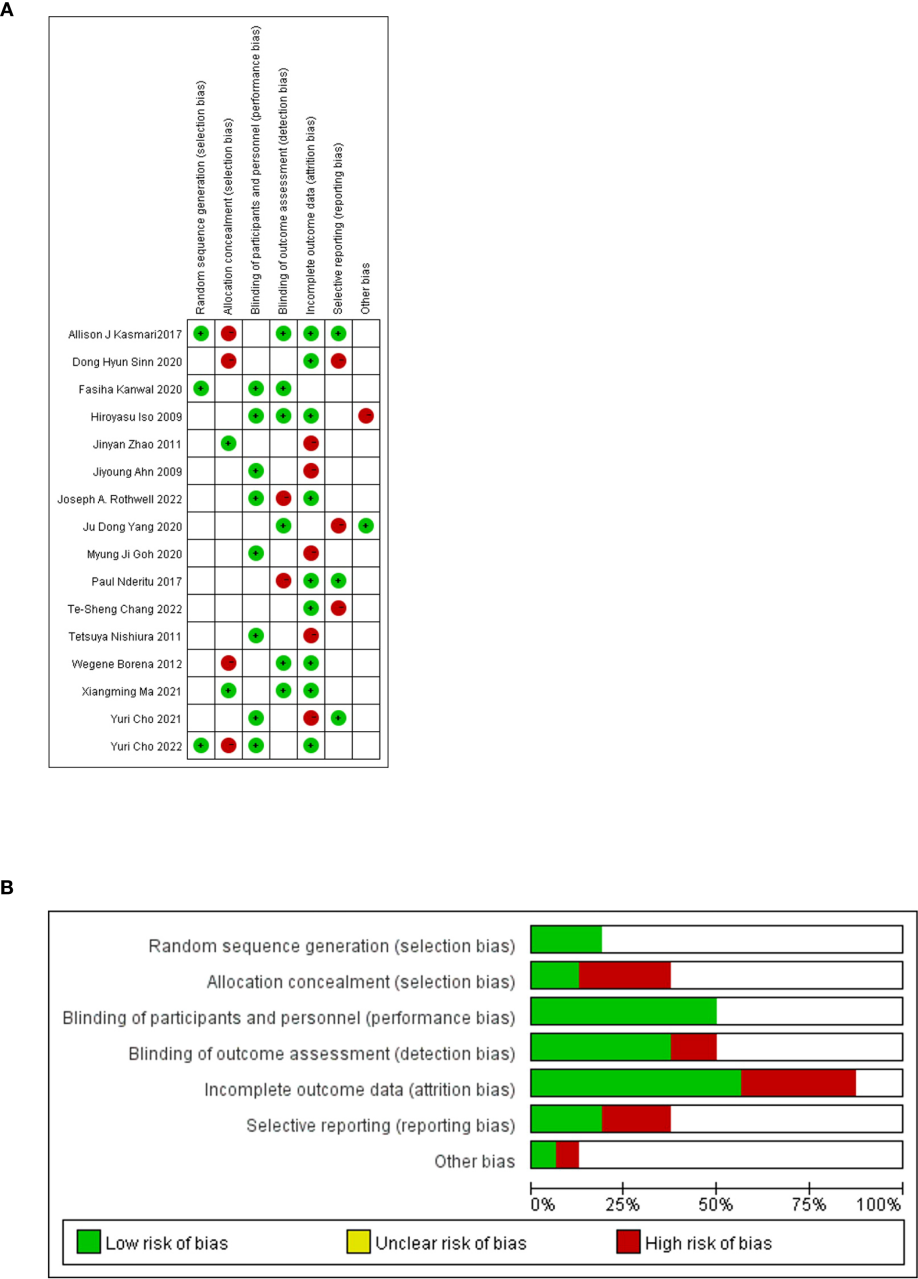
Quality evaluation of the included literature by QUADAS-2 based on Revman software. **(A)** Risk of bias graph. **(B)** Risk of bias summary.

Using RevMan software, the evaluation of the 16 papers incorporated in this study was conducted by our team to assess their quality (**Figures 3**). The majority of these studies demonstrate medium to high quality, indicating their reliability. However, six studies exhibit susceptibility to attrition bias due to incomplete data, while four papers are prone to selective bias, lacking explanations regarding the potential predictability of distribution results by subjects and researchers.

### 3.4 Publication bias

To assess potential publication bias within our analysis, we employed the Deeks funnel chart asymmetry test, plotting the effect value-to-standard error ratio for each study. The funnel plot indicated slight to moderate asymmetry among certain variables. Utilizing Egger regression asymmetry and Begg rank correlation tests, we observed slight asymmetry in studies related to TC, albeit this finding lacked confirmation through the Begg rank correlation test (**Supplementary Figure 1**).

### 3.5 Sensitivity analysis

To gauge the strength and reliability of our primary findings, sensitivity analyses were undertaken as part of our research methodology, systematically excluding individual studies from the meta-analysis. For serum cholesterol and low-density lipoprotein meta-results, we applied a leave-one-out approach, revealing that the outcomes remained generally stable and robust upon exclusion of each study one at a time. However, the results concerning serum triglycerides were influenced by a single study, and upon its removal from the analysis, a change in the results was observed. Specifically, it was observed in our analysis that initially, elevated levels of serum triglycerides were linked to a protective effect against the occurrence of liver cancer, but this association became statistically insignificant after excluding the study by Yuri et al. (HR = 0.69, 95% CI: 0.45, 1.04, I2 = 94%) (**Supplementary Figure 2**).

## 4 Discussion

### 4.1 Main findings

In the course of our research, we performed a thorough analysis that amalgamated evidence pertaining to the risk of liver cancer and serum lipid levels. Through the meta-analysis, an adverse connection between increased blood cholesterol concentrations and the likelihood of hepatic carcinoma was unveiled. Moreover, serum low-density lipoprotein, elevated triglycerides, and dyslipidemia exhibited associations with reduced liver cancer risk, albeit with considerable variability between studies. Conversely, no significant correlation emerged between serum HDL and liver cancer risk. Except for serum triglyceride, other findings remained generally stable and consistent throughout sensitivity analyses.

Lipids, comprising diverse biomolecules, serve critical functions in cellular energy storage, structural integrity, and signal transduction. In clinical contexts, plasma lipids hold extensive associations with various diseases, prompting routine evaluations (31). Of particular concern is the pivotal role lipids play in tumor initiation and progression (32). Rapidly dividing cancer cells rely on a continuous supply of lipids for cell membrane formation and protein modification. Conversely, slowly proliferating cancer cells augment lipid content to fortify signal transduction pathways and evade apoptosis (32). Prior research indicates that cancer patients frequently exhibit ​low serum levels of total cholesterol, LDL, and HDL, with compelling evidence linking ​this hypolipidemic profile​ to cancer pathogenesis and progression (33).

Across various cancer types, including liver, hematologic, intestinal, lung, prostate, head, and neck cancers, low serum cholesterol levels have been frequently observed. Additionally, studies conducted on cohorts indicate a connection between diminished serum cholesterol levels and the overall risk of developing cancer (34). In our investigation, we propose a negative correlation between the probability of hepatic carcinoma and the concentrations of blood cholesterol, prompting the need for further investigations into its specific mechanisms. Moreover, extensive research on different lipoprotein subfractions—such as LDL, HDL, and VLDL—has consistently shown reduced serum cholesterol, HDL, and LDL levels alongside increased serum triglyceride levels, a prevalent and consistent trend warranting attention in understanding cancer risk factors (31).

Our findings suggest that elevated serum levels of LDL serve as protective factors against the risk of liver cancer. Contrarily, several studies have indicated that low levels of serum LDL may contribute to the development of liver cancer—this is further supported by observations where prostate cancer cell growth rates surged in LDL-rich mediums (35). The uptick in LDL uptake by cancer cells might hinder their clearance from circulation, potentially contributing to lowered serum LDL in cancer patients (36). The decrease in serum LDL among cancer patients also points to an intriguing inference: lipid metabolism, influencing cell apoptosis through membrane regulation and enzyme activation (37), particularly relates to specific programmed cell death mechanisms like iron death (38), facilitated by iron-dependent membrane lipid peroxidation (39). Replacing saturated fat (SFA) with unsaturated fat in dietary improvement has shown promise in reducing LDL cholesterol and elevating HDL cholesterol, enhancing overall lipid profiles (40). However, monounsaturated fatty acids (MUFA) within unsaturated fats don’t initiate lipid peroxidation, unlike polyunsaturated fatty acids (PUFA), which promote this process (41). Melanoma cells spreading via the lymphatic system show resistance to iron death, attributed to their uptake of MUFA from the lymphatic environment into their membranes (42). This suggests a potential link between excessive MUFA intake before liver cancer onset and the prevention of lipid peroxidation-induced iron death in cancer cells, potentially promoting liver cancer occurrence. Despite serum LDL level declines, the precise mechanism remains unclear, yet the accumulation of monounsaturated lipids appears to shield liver cancer cells from lipid peroxidation. Moreover, scant contradictory studies indicate an increased cancer risk linked to high-density lipoprotein levels, aligning with our research findings (43).

Examining serum triglycerides, an Italian comparative study on blood lipid and cancer presented conflicting results regarding serum TG levels across different tumor types. However, overall, cancer patients tended to exhibit relatively high blood lipid characteristics (44). Our meta-analysis findings denote serum TG as a protective factor against liver cancer risk. While the limited and contradictory literature included demonstrates an overarching negative correlation trend, the specific mechanism warrants further exploration. Shifting our focus to dyslipidemia and its connection with the risk of developing liver cancer, current guidelines assert a significant association is observed between nonalcoholic liver disease (NAFLD) and metabolic risk factors, particularly emphasizing obesity, type 2 diabetes (T2DM), and dyslipidemia (45). Mounting evidence links NAFLD to an increased risk of HCC, suggesting a potential role for dyslipidemia in promoting liver cancer risk (46). However, our research surprisingly reveals dyslipidemia not as a promoter but as a protective factor against liver cancer—an unexpected outcome. Upon revisiting the literature, inadequate inclusion volume and varying dyslipidemia definitions emerged as key reasons for the inconsistent data, including differing baseline levels and diverse exclusion criteria in the included studies. Hence, a more in-depth and thorough examination and analysis are required to interpret the results related to dyslipidemia.

The interplay of various environmental and genetic factors profoundly shapes human plasma lipid profiles (47), underscoring the importance of discerning the biological relevance between distinct lipid components and liver cancer. Consideration of confounding factors affecting serum lipid profiles is pivotal in preventing overlooked risk factors from confounding analyses and yielding perplexing outcomes. Our findings introduce novel evidence for liver cancer, pinpointing a correlation between high cholesterol content (≥200mg/dL) and decreased liver cancer risk. Yet, the limited number of studies warrants cautious interpretation of this result. First of all, Inconsistencies in the relationship between cholesterol and liver cancer risk may stem from varied lipid acquisition modes adopted in different cohort studies, influencing plasma lipid profiles. Reports of diverse plasma lipid profiles among liver cancer patients hint at potential metabolic strategies adopted by cancer cells, necessitating further investigation into plasma lipid’s mechanisms and significance in liver cancer progression. The connection between serum lipids and liver cancer involves a role played by genetic susceptibility. Moreover, undiagnosed liver conditions like cirrhosis and nonalcoholic fatty liver disease (NAFLD) could impact cholesterol metabolism, potentially intensifying the negative correlation with the risk of liver cancer. For example, an association is noted between persistent hepatitis B virus infection and an elevated likelihood of hepatic carcinoma alongside reduced cholesterol levels, blurring the qualitative distinction between blood cholesterol levels and the risk of liver cancer. In this scenario, elevated cholesterol concentrations may indicate facets of hepatic function rather than serving as a direct pathogenic element in the likelihood of hepatic carcinoma (48). Then, Of the 16 studies incorporated in our meta-analysis, excluding only 9 participants based on the presence of cancer or cardiovascular disease restricts our capability to thoroughly assess the influence of undiagnosed liver diseases on the outcomes we have obtained. Moreover, our outcomes might be influenced by the heightened use of cholesterol-lowering medications, specifically statins, among individuals with elevated cholesterol levels (49). The utilization of statins has been linked to a decreased likelihood of developing liver cancer (50), yet only 2 studies among those we reviewed excluded statin users. The majority of inquiries into the relationship between serum lipids and liver cancer do not incorporate adjustments for the utilization of statins. Although the utilization of statins was uncommon prior to 1990, the available baseline data from that timeframe were restricted. Regardless, the outcomes indicated a reciprocal connection between the concentrations of blood cholesterol and the likelihood of hepatic carcinoma (ORH/L=0.60, 95% CI: 0.46, 0.78, n=3, I^2^ = 81.2%). Clearly, further data on statin use is warranted to comprehensively assess their influence on our results.

### 4.2 Influences

Our meta-analysis offers fresh insights into the relationship between serum cholesterol, HDL, LDL, TG, dyslipidemia, and the risk of liver cancer. Surprisingly, our results unveiled an unforeseen opposite correlation between the concentrations of blood cholesterol and the likelihood of hepatic carcinoma, prompting the necessity for additional exploration to clarify this unanticipated association. However, the limited research available on other lipid subtypes like HDL and LDL underscores the need for more comprehensive studies in these areas.

### 4.3 Strengths and limitations

Our research offers notable advantages. Firstly, we conducted category and subgroup analyses, enhancing the depth of our investigation. Secondly, in the majority of serum lipid studies (15 out of 16), at the initiation of the cohort, blood specimens were gathered and subsequently scrutinized for a period exceeding 5 years. This approach potentially mitigates concerns about reverse causality, strengthening the reliability of our findings. Most importantly, in the latest published analysis of the association between lipids and liver cancer risk (10), we obtained different results using different information sources. We found that LDL was negatively correlated with liver cancer risk, and we made a more detailed explanation for this result, which was not reflected in the latest published literature.

Some noteworthy limitations should be considered. Firstly, our inclusion of both prospective and retrospective studies might affect the internal validity of our findings. Secondly, the relatively limited number of studies may hamper statistical power, especially in detecting associations and potential publication bias—highlighted in instances such as low-density lipoprotein and dyslipidemia, which involved only a few retrieved studies. Thirdly, substantial inter-study heterogeneity in the relationship between serum lipids and liver cancer was observed, where, given the restricted quantity of studies available, a comprehensive discussion on the sources of heterogeneity for certain serum lipids was not undertaken. Fourth, our analysis is confined by the original research’s limitations. For instance, dietary information gathered primarily through Food Frequency Questionnaires (FFQ) might be prone to measurement errors. Fifth, the bulk of inquiries lacked details on hepatitis B virus (HBV) and hepatitis C virus (HCV), constraining our ability to assess their influence on the outcomes, although it is probable that their impact on the recorded connection between dietary habits and hepatic carcinoma is negligible (51). Finally, in our ongoing meta-analysis, 7 out of 9 studies concentrating on hepatic conditions regarded hepatic carcinoma as their principal result, with 1 specifically focusing on liver cancer risk. Additionally, among the 7 serum lipid studies, 4 prioritized liver cancer as the primary outcome, while 2 concentrated on hepatocellular carcinoma risk. Given statins’ estimated 15-30% HCC risk reduction and 20-40% LDL-lowering effect, we calculate that unadjusted statin use could theoretically attenuate true LDL-HCC associations by ≤12% (using Bross formula). This does not invalidate our primary finding. Consequently, our findings predominantly pertain to HCC, and we faced limitations in thoroughly evaluating the potential etiological heterogeneity among various subtypes of HCC.

## 5 Conclusions

The meta-analysis we conducted unveiled a noteworthy correlation indicating that higher levels of serum cholesterol are linked to a diminished likelihood of hepatic carcinoma. Moreover, heightened concentrations of LDL and triglycerides were also linked to a reduced probability of contracting hepatic carcinoma, although substantial heterogeneity among studies was observed. Further investigation is necessary to elucidate the underlying mechanisms behind these associations.

## Data Availability

The original contributions presented in the study are included in the article/**Supplementary Material**. Further inquiries can be directed to the corresponding author.
