# Supplementary figures and images for: Inverse association between serum lipid profiles and hepatocellular carcinoma risk: a meta-analysis of epidemiological studies
Source: Front Oncol. 2025 Oct 22;15:1644677. doi: 10.3389/fonc.2025.1644677 (PMC12585945; doi:10.3389/fonc.2025.1644677)

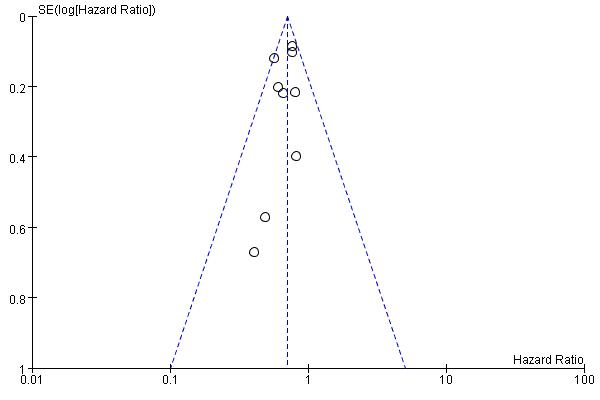

Supplement: Supplementary file 1 [file Image1.jpg]

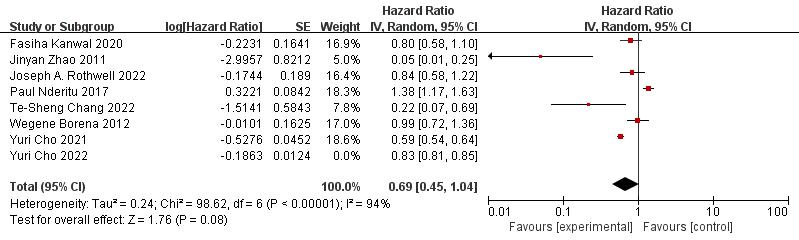

Supplement: Supplementary file 2 [file Image2.jpg]
